# Immobilization in external rotation vs internal rotation after shoulder dislocation: A systematic review and meta-analysis protocol

**DOI:** 10.1097/MD.0000000000016707

**Published:** 2019-08-09

**Authors:** Xin Cui, Long Liang, Hongyan Zhang, Jing Zhao, Yongyao Li, Hao Cheng, Shiheng Wang, Yue Zhang

**Affiliations:** a Institute of Basic Research in Clinical Medicine, China Academy of Chinese Medical Sciences; b Wangjing Hospital, China Academy of Chinese Medical Sciences, Beijing; c Guangzhou University of Traditional Chinese Medicine, Guangzhou; d China Institute for History of Medicine and Medical Literature, China Academy of Chinese Medical Sciences, Beijing; e Longhua Hospital, Shanghai University of Traditional Chinese Medicine, Shanghai, China.

**Keywords:** external rotation, internal rotation, protocol, shoulder dislocation, systematic review

## Abstract

**Background:**

Dislocation of shoulder joint is the most prone to occurrence in all joints of human body, which is common in young people and has a high recurrence rate. It is mainly treated by conservative treatment. External rotation and internal rotation fixation are 2 common conservative therapies in clinical practice. Therefore, we conduct this systematic review and meta-analysis to evaluate the efficacy and safety of the 2 treatments.

**Methods:**

Nine electronic databases, PubMed, Web of Science, Embase, Cochrane Library, Cochrane Central Register of Controlled Trials (CENTRAL), and ClinicalTrials.gov, CNKI, Wanfang Database and VIP Database, will be searched to find and include randomized controlled trials that meet inclusion criteria. RevMan5.3 will be used for data analysis and synthesis in this study. Subgroup analysis and sensitivity analysis will also be performed if necessary. In addition, GRADE will be used in the evaluation of evidence hierarchy.

**Results:**

This study will analyze and integrate the original evidence so far for clinical efficacy and safety of immobilization in external rotation and internal rotation on shoulder dislocation.

**Conclusion:**

The conclusion of this study will conclude higher evidence and suggestions for the treatment of shoulder dislocation, so as to further guide clinical decision making.

**PROSPERO registration number:**

CRD42018106030.

## 1 Introduction

With the largest range of motion and relatively low stability, shoulder joint is the most prone to dislocation.^[1,2]^ Shoulder dislocation, nearly 90% to 95% of anterior dislocation,^[2,4]^ accounts for about 45% of all dislocation in orthopedics,^[3]^ which is predominant in young people.^[1,5]^ It is more than triple the incidence rate for women.^[6]^ According to Kirkley's study, the occurrence of 1st shoulder dislocation is 8 to 8.2/100,000 people/year.^[7]^ Shoulder dislocation often occurs when the arm is forced to abduct, rotate, and overextend, which can lead to the humerus head out of the shoulder joint.^[8–14]^

The treatment of shoulder dislocation can be divided into operation and conservative therapy. Surgical treatment is used only for complex dislocation or failure of conservative reduction.^[15,16]^ Therefore, conservative treatment is the main measure. Conservative treatment is usually followed by internal or external rotation fixation after reduction.^[16]^ Some studies have shown that internal rotation fixation can reduce the recurrence rate compared with external rotation fixation,^[17–19]^ while others have the opposite results.^[20]^ Consequently, it is necessary to conduct a systematic review of immobilization in external rotation vs internal rotation on shoulder dislocation with the increasing of related studies in recent years. This study will be performed to analyze and integrate the existed evidence regarding efficacy and safety of immobilization in external rotation and internal rotation on shoulder dislocation, which can conclude higher evidence for the treatment of shoulder dislocation.

## 2 Methods

### 2.1 Study registration

This protocol has been registered in the international prospective register of systematic reviews (PROSPERO), and the registration number is CRD42018106030. Available at: http://www.crd.york.ac.uk/PROSPERO/display_record.php?ID=CRD42018106030. The steps of this protocol will follow the Preferred Reporting Items for Systematic Review and Meta-analysis Protocols (PRISMA-P) statement guidelines.^[21]^ Since this study is a secondary literature study based on randomized controlled trials (RCTs), no ethical approval and patient consent are required.

### 2.2 Inclusion criteria for study selection

#### 2.2.1 Type of studies

We will only include RCTs. Retrospective studies, review, case reports, cohort studies, and experimental studies will be excluded. There are no restrictions on languages.

#### 2.2.2 Type of participants

We will include studies that the patients must be definitely diagnosed as shoulder dislocation, not limited by gender, ethnicity, nationality, primary disease, or clinical stage, which was based on imaging diagnostic criteria.

#### 2.2.3 Type of interventions

We will include the studies that immobilization in external rotation is considered as an intervention in the treatment group, while immobilization in internal rotation is included in the control group.

#### 2.2.4 Type of outcome measurements

##### 2.2.4.1 Primary outcomes

Recurrence rate will be defined as the primary outcome to assess the frequency of recurrence of dislocation.

##### 2.2.4.2 Secondary outcomes

1. Compliance rate, measured by any instrument.
2. Adverse events, measured by any instrument.
3. Shoulder function, measured by the Western Ontario Shoulder Instability.

### 2.3 Search strategy

Relevant literature were retrieved using multiple online databases including the PubMed, Web of Science, Embase, the Cochrane Library, the Cochrane Central Register of Controlled Trials (CENTRAL), ClinicalTrials.gov, the Chinese National Knowledge Infrastructure Database (CNKI), Wanfang, and VIP Database. No limits were imposed on the dates, types, and statuses of the publications eligible for inclusion. The key terms used in the searches were: “shoulder dislocation,” “glenohumeral dislocation,” “glenohumeral subluxation,” “bankart lesion,” “external rotation,” “internal rotation,” “fixation,” “immobilisation,” “reduction.” Different search strategies were used for the Chinese and foreign language databases. In addition, the reference lists of previously published systematic reviews on the subject of external rotation and internal rotation immobilization for the treatment of shoulder dislocation were manually examined for pertinent studies.

### 2.4 Selection of studies

Two reviewers independently read the title and abstract of the literature, and screened the documents according to inclusion and exclusion criteria. When they are uncertain to determine whether to exclude, we will read the full text to identify the studies that need to be included.

### 2.5 Data extraction

**Figure 1:**
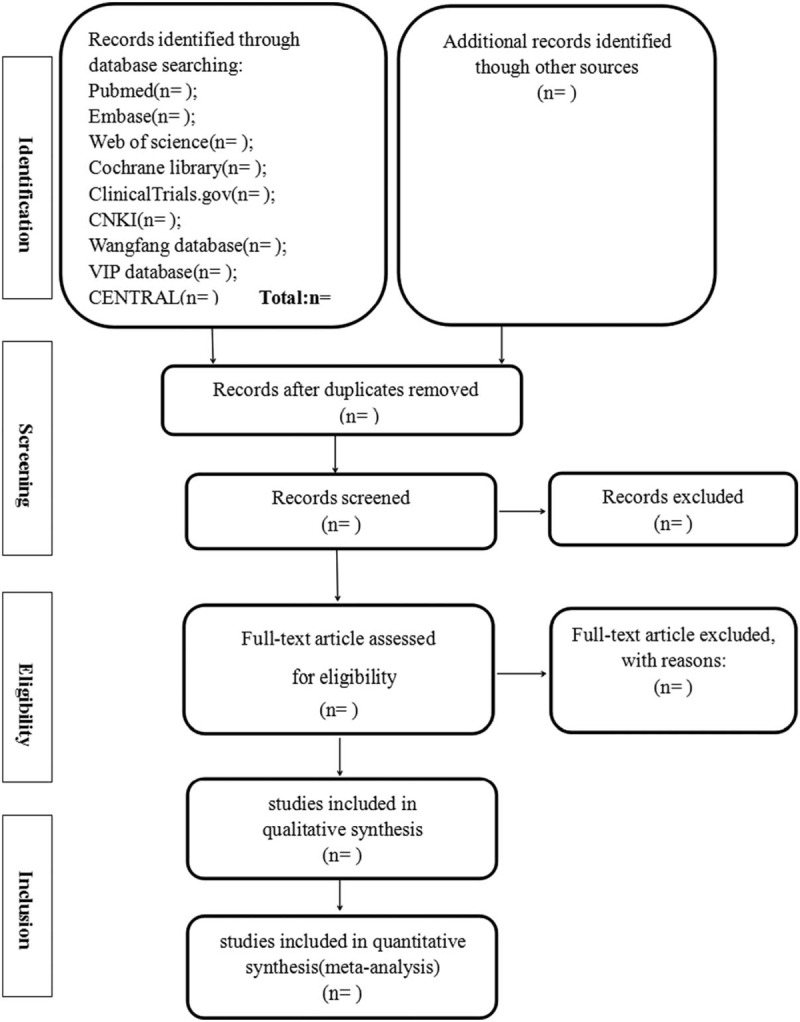
Flow diagram of study selection and screening process.

The following data will be independently extracted by 2 authors: the name of 1st author, year of publication, country, number of patients under total disc replacement and lumbar fusion, sample size, age, gender of patients, disease course, follow-up duration. When relevant data have not been reported, we will contact the authors by email or in other ways to attempt to obtain the missing information. The review authors will resolve any disagreements by discussion, including input from a 3rd independent review author if required. The flow diagram (Fig. 1) will be used to show the details of the study selection process.

### 2.6 Assessment of risk of bias

Two authors will assess the methodological quality of the included studies using the criteria outlined in the Cochrane Handbook for Systematic Reviews of Interventions 5.1.0.^[22]^ Two authors will also compare the results and will discuss any differences until agreement is reached. The domains to be assessed will include: random sequence generation, allocation concealment, blinding of participants and personnel, blinding of outcome assessment, incomplete outcome data, selective reporting, and other bias.

For other sources of bias, 2 aspects have been identified: trials stopped early owing to some data-dependent processes; baselines extreme imbalanced.

### 2.7 Measures of treatment effects

The outcomes of interest will include dichotomous data and continuous variables. Dichotomous data will be expressed as the risk ratio (RR), and mean difference (MD) will be used to assess differences in the continuous outcomes between the groups. Also, standardized mean difference (SMD) will be chosen if the clinical outcomes are the same, but have been measured using different methods in different trials. The corresponding 95% confidence interval (CI) for each parameter will be computed for the treatment group vs the control group. If quantitative synthesis is not appropriate, descriptive review will be selected.

### 2.8 Assessment of heterogeneity

Statistical heterogeneity across the included studies will be examined using the *I*^2^ statistic, with an *I*^2^ > 50% regarded as being indicative of the possibility of statistical heterogeneity, resulting in the selection of a random-effects model for the computation of MD or SMD with its corresponding 95% CI. Otherwise, no obvious heterogeneity will be considered to be present in the included studies for values of *I*^2^ < 50%, in which case the fixed-effects model will be selected to generate the MD or SMD with its corresponding 95% CI.

### 2.9 Assessment of publication bias

If more than 10 original studies are included, funnel plots will be made according to the data of the included studies to observe publication bias. If the funnel plot is asymmetric, it indicates publication bias. We will discuss the sources and explanations of bias.

### 2.10 Data synthesis

A forest plot for each parameter will be constructed to illustrate the weight ratio of each incorporated study. All statistical analyses will be carried out using the RevMan5.3 software, and the significance threshold will be a 2-sided *P* < .05.

### 2.11 Sensitivity analysis

To evaluate the sensitivity of the meta-analysis, studies will be excluded one by one, and the differences of the combing effects before and after exclusion will be compared, and if the pooled outcomes are found to have been reversed after the exclusions, the outcomes may be unstable.

### 2.12 Subgroup analysis

When heterogeneity is high, if the necessary data are available, subgroup analyses will be conducted for different comparators separately. In addition, if the expected efficacy is not observed in all the subjects, subgroup analysis could help us show whether the treatment is effective in some specific subgroups. At the same time, subgroup analysis can also help us to show whether the therapeutic effect is better in particular subjects if it is found to be effective in all subjects.

### 2.13 Grading the quality of evidence

The Grading of Recommendations Assessment, Development, and Evaluation (GRADE) method is used to evaluate the quality of evidence for each outcome of meta-analysis. The GRADE Working Group recommended that the quality of evidence can be classified into four levels: high (++++), moderate (+++), low (++), and very low (+). Evidence quality is generally judged on the basis of risk of bias, inconsistency, indirectness, inaccuracy, and publication bias. We can evaluate it on this page: https://gradepro.org/.

## 3 Discussion

This systematic review and meta-analysis will integrate the latest and most comprehensive original clinical research evidence in this field. It mainly evaluates the efficacy and safety of external rotation and internal rotation fixation in the treatment of shoulder dislocation, which by assessing the outcome such as recurrence rate, compliance rate, adverse events, and other outcomes.

At the same time, this study has been registered on the international prospective register of systematic reviews (PROSPERO), which will make the procedures and results of this study more transparent and further improve its credibility. In addition, GRADE evidence-based evaluation method will be used to evaluate the quality level of original research evidence in this study, which can contribute to the transformation of study results and the formation of guideline. We hope that high-level evidence for the treatment of shoulder dislocation can be concluded in this study, which can provide recommendations for clinicians to make decisions about this disease.

## Author contributions

**Conceptualization:** Xin Cui, Long Liang.

**Data curation:** Xin Cui, Hongyan Zhang, Yue Zhang.

**Methodology:** Long Liang, Jing Zhao.

**Resources:** Hongyan Zhang, Yongyao Li, Hao Cheng.

**Software:** Xin Cui, Hongyan Zhang, Shiheng Wang.

**Supervision:** Jing Zhao.

**Writing – original draft:** Xin Cui, Long Liang.

**Writing – review & editing:** Xin Cui, Long Liang, Jing Zhao, Yongyao Li, Hao Cheng.

Xin Cui orcid: 0000-0003-2257-6724.
